# Exploring the feasibility of using evidence-based feeding practices to promote children’s healthy eating in holiday clubs

**DOI:** 10.1017/S1368980023002276

**Published:** 2023-12

**Authors:** Natasha Bayes, Carolynne Mason, Emma Haycraft, Clare E Holley

**Affiliations:** 1 School of Sport, Exercise and Health Sciences, Loughborough University, Epinal Way, Loughborough, Leicestershire, LE11 3TU, UK; 2 Department of Psychology, University of Liverpool, Eleanor Rathbone Building, Bedford Street South, Liverpool L69 7ZA, UK

**Keywords:** Food insecurity, Feeding practices, Eating behaviour, Holiday clubs, Modelling

## Abstract

**Objective:**

Encouraging healthy eating is a public health priority in the United Kingdom (UK), given the high prevalence of poor diet and overweight/obesity among school-aged children. Holiday clubs are organisations providing childcare and activities during the school holidays and frequently provide food to children at risk of food insecurity, primarily through government-funded programmes like the Holiday Activities and Food programme. However, the research suggests that holiday clubs could do more to maximise opportunities to promote children’s healthy eating by using evidence-based feeding practices.

**Design:**

During August–September 2020, video-based interviews were conducted exploring staff perceptions of the feasibility of using four evidence-based feeding practices to promote children’s healthy eating: modelling; involvement in food choice; involvement in food preparation and cooking and involvement in meal planning. Feasibility was assessed using four dimensions of a feasibility framework (acceptability, demand, practicality and implementation).

**Setting:**

UK holiday clubs.

**Participants:**

Twenty-five staff actively involved in delivering UK holiday clubs (project leaders, coordinators, cooks and coaches/youth workers).

**Results:**

Staff generally reported good acceptability (dimension 1) and demand (dimension 2) for the feeding practices. However, the practicality (dimension 3) of using the practices was dependent on various factors (logistics, resources, staff readiness, children, peers and parents). Promisingly, in the fourth feasibility dimension (implementation), staff provided numerous practical solutions to overcome these barriers.

**Conclusions:**

Evidence-based feeding practices can be implemented in numerous ways and are therefore generally feasible in holiday clubs. Holiday clubs should be empowered to use evidence-based feeding practices through training resources, sharing networks and provision of sustainable funding.

The UK is the sixth richest country in the world^(1)^, yet it has the highest rate of food insecurity in Europe^(2)^. Current estimates suggest that during February and August 2021, almost 10 % of UK households experienced food insecurity, equating to 5·2 million adults and 2·5 million children^(3)^.

Children are recommended to consume at least five portions (approximately 400 g) of fruit and vegetables per day^(4)^. Despite this, only 19 % of children in the UK aged 5–15 years consume the recommended intake of fruit and vegetables^(4)^ and so providing opportunities for children to consume fruit and vegetables is important for encouraging a healthy diet and protecting them against a range of diet-related diseases. Food insecurity has been widely linked to diet-related diseases such as diabetes, cancer, CVD, undernutrition and obesity^(5)^. These outcomes are understandably prevalent among food insecure families because the experience of food insecurity often forces families to make difficult food-related decisions when food is scarce or food quality is compromised. For example, families who are food insecure often have little choice but to make cheaper, less nutritious foods available in the home environment^(6)^ and often have fewer opportunities to engage in health-promoting parent–child interactions (e.g. can be more likely to pressure children to eat to ensure children are fed and to avoid food waste)^(7)^. Exposure to this environment can inadvertently contribute to poorer long-term health and dietary outcomes for children, such as CVD and cancer^(8)^, as well as all-cause mortality^(9)^, as it can have counterproductive effects on children’s eating behaviour such as contributing to selective food preferences^(10)^, and it can disrupt children’s ability to self-regulate energy intake^(11)^. Understanding ways to provide opportunities for children in families at risk of food insecurity to consume fruit and vegetables within their means is therefore important for supporting them to eat a nutritious diet.

One factor known to influence children’s eating behaviours and dietary intake is caregiver feeding practices. A child’s caregiver can be anyone carrying out care or parental responsibilities towards a child (e.g. parent at home, a teacher at school, a staff member in nursery or childcare settings). Feeding practices are the interactions between caregiver and child around food and mealtimes which shape the child’s eating behaviour. A wide range of feeding practices exist, and they are typically characterised as positive (where positive eating behaviours are shaped in children from the feeding practice) or maladaptive (where negative eating behaviours are shaped in children from the feeding practice). For example, modelling is typically a positive feeding practice when it includes demonstrating healthy eating to children through behaviours such as verbalising positive statements about the food they are eating. Modelling healthy eating behaviours is associated with children’s positive eating behaviours such as increased consumption of fruit and vegetables^(12)^, encouraging children to try new foods^(13)^ and less food fussiness^(14)^. Another positive feeding practice is involvement, where children are included in food-related activities such as meal planning and food preparation. Involvement is associated with children’s positive eating behaviours such as increased consumption, liking and willingness to taste novel foods^(15)^, greater enjoyment of food and lower food fussiness^(16)^. Consequently, many eating behaviour interventions implement positive feeding practices to positively shape children’s eating behaviours. For example, one study involved UK food insecure primary school children in a school-based cooking intervention to improve their cooking confidence and vegetable consumption, which resulted in significant post-intervention improvements^(17)^.

There are numerous settings that provide meaningful opportunities to promote children’s healthy eating, such as homes, schools and childcare (including breakfast, after-school and school holiday programmes)^(18)^. Children spend extensive periods of time in these settings, and therefore have a significant number of their food and mealtime experiences within them^(18)^. Holiday clubs are organisations providing childcare and activities to children during the school holidays^(19)^. Holiday clubs are reported to vary in how they are funded, what settings they run in, what age groups and volumes of children they provide for and what frequency and type of provision they offer^(19,20)^. However, holiday clubs provide a range of services that usually include food, enrichment opportunities (e.g. sport and physical activity) and information and education^(19)^. This means that holiday clubs are a setting that are well-placed to promote children’s healthy eating, as they increasingly provide free food to children at risk of food insecurity^(21)^, particularly as part of the Holiday Activities and Food (HAF) programme. The HAF is a national UK government funded programme aiming to provide food (to promote healthy eating), activity (to promote exercise) and enrichment (to promote socialising, expression and creativity) opportunities through new and existing UK holiday clubs to children who are free school meal (FSM) eligible^(22)^. The programme began in 2018 with £2 million for programme piloting^(23)^, funding seven holiday club providers^(24)^ and reaching approximately 18 000 children at risk of food insecurity during the 2018 summer holidays^(25)^. The programme has since expanded to £220million to fund holiday clubs in 151 local authority areas during the 2021 school holidays^(26)^. Research suggests that holiday clubs provide multiple benefits to children, including alleviating hunger^(21)^ and promoting healthy eating. Holiday clubs can promote healthy eating by providing access to a variety of foods, using innovative strategies to promote tasting of novel foods and promoting positive social experiences around food^(27)^. This reinforces that holiday clubs are an ideal setting for promoting healthy eating to children and for providing health-promoting opportunities that FSM eligible children may miss out on during the summer holidays (e.g. healthy food availability, engaging in cooking activities).

As positive feeding practices are known to improve children’s eating behaviour outcomes, holiday clubs may benefit from maximising the opportunities to use these feeding practices with children. However, only a few recent studies exist in relation to the use of feeding practices by staff in holiday clubs^(20,28,29)^. These studies suggest that some holiday clubs are using evidence-based feeding practices^(20)^, such as providing opportunities to involve children in food-related activities^(29)^ and providing nutrition education^(28)^. Although promising, these studies also highlight that the use of evidence-based feeding practices varies across holiday clubs^(28)^, and there is a need for holiday clubs to identify opportunities to increase the use of positive feeding practices (where positive eating behaviours are shaped in children from these feeding practices)^(20)^. It is therefore salient to explore staff perceptions of the theoretical feasibility of using evidence-based positive feeding practices in holiday club settings.

Feasibility studies are beneficial when little is known about the potential implementation of behavioural or social interventions, to generate understanding of what factors might impact their delivery^(30)^. Qualitative methods are increasingly used to assess feasibility with relevant target populations and settings^(30)^, to gain in-depth views and perspectives from key stakeholders. This qualitative study explored the feasibility of holiday club staff using four evidence-based feeding practices with children in holiday clubs: modelling and three variations of involvement (involvement in food choice; involvement in food preparation and cooking; involvement in meal planning). These feeding practices were selected because of the benefits they have on children’s eating behaviour^(15–17)^, and because the results from a previous study^(20)^ reported the importance of staff as role models to children, and the holiday club environment being an ideal setting for involving children in meaningful food-related activities. With this in mind, modelling and involvement may be particularly relevant feeding practices to use in holiday club contexts when feeding children. The current study provides an opportunity to test these assumptions and identify the extent to which modelling and involvement are feasible in holiday clubs.

## Methods

### Philosophical position

This study was implemented within an interpretivist research paradigm. The study adopted a qualitative multi-method approach, using vignettes alongside semi-structured video/telephone-based interviews with club staff to explore the feasibility of using four evidence-based feeding practices in holiday clubs.

This study received ethical approval from Loughborough University's Ethics Committee (ref: SSEHS-1644).

### Feasibility framework

The study design and structure of the data analysis were guided by an existing feasibility framework^(31)^, which has been previously utilised to guide the design of various public health interventional research^(32)^. The framework includes eight dimensions, four of which were applied to this study:

1. Acceptability: Exploring the extent to which a new idea (or programme, process or measure) is judged as suitable, satisfying or attractive to programme deliverers and/or programme recipients;
2. Demand: Exploring the extent to which a new idea is likely to be used;
3. Practicality: Exploring the extent to which an idea can be carried out with intended participants using existing means, resources and circumstances and without outside intervention;
4. Implementation: Exploring the extent to which a new idea can be successfully delivered to intended participants in some defined, but not fully controlled, context.

Dimensions 1 and 2 were selected because little is currently known about the current attitudes or actions related to the use of feeding practices in the holiday club context, which the acceptability and demand feasibility dimensions tap into. Dimensions 3 and 4 were selected because there are a range of factors (e.g. resource and time constraints) that can facilitate or hinder the implementation of public health interventions, which the practicality and implementation feasibility dimensions tap into. As such, previous research suggests that holiday programmes have experienced resource constraints^(33)^, and the feasibility dimensions explored in this study will help to identify whether issues such as resource constraints hinder the feasibility of using evidence-based feeding practices in holiday clubs.

### Participants

Participants were staff who were actively involved in the delivery of UK holiday club provision during the 2020 school summer holidays. Participant inclusion criteria were: 1. being age 18 or over; 2. conducting a paid and/or voluntary role within the club; 3. working in clubs that involved mostly primary school aged children (e.g. ages 5–11 years) and provided food and 4. working in clubs with high proportions (50 %+) of FSM eligible children attending the club. The age criterion was imposed to this study because 1. caregiver influences on children’s eating behaviour lessen as children move into adolescence, and interventions to improve children’s eating behaviour outcomes have been shown to be more successful among younger children compared to adolescents^(34)^ and 2. complementing point 1; while a large proportion of holiday clubs engage children from the 5–12 age range, the criterion helped to avoid recruiting holiday clubs which exclusively engage adolescent age children. The FSM eligibility criterion was imposed as the study sought to engage clubs providing opportunities for children at risk of food insecurity, and FSM eligibility is an indicator of deprivation and increased risk of food insecurity.

A total of twenty-five club staff from twenty four different clubs across nine UK regions engaged in the study (Table 1). The clubs were run in various settings, including community centres, sport centres and schools.

**Table 1:** Club staff regional localities

Region	*n*
East Midlands	7
Yorkshire and the Humber	4
South East	4
South West	3
North West	2
West Midlands	2
London	2
East of England	1
Wales	1

### Recruitment and data collection

Desk-based research was conducted to develop a database of charity and government holiday club programme providers. Organisations in the database (*n* 40) were contacted requesting their support with recruitment of club staff. Fifteen (nine local, six national) organisations overseeing UK holiday club programmes supported recruitment, by circulating a study poster via social media (Twitter and Facebook) and/or email to clubs implementing their programme. Interested club staff were directed to an online survey to read a full participant information sheet, complete a consent form, then provide their name, contact details, club information (length of employment, role, average number/age of child attendees) and demographic questions (age, gender, ethnicity and nutrition qualifications).

Participants expressing their willingness to participate (*n* 27) were contacted to organise a video-based interview, 25 of whom responded. Video-based interviewing was selected for its screen-sharing functionality, which enabled study vignettes to be viewed and discussed during the interview, when the global COVID-19 pandemic limited in-person interactions. Telephone interviews were conducted with two participants due to issues with internet connectivity and/or absence of relevant technology.

Interviews lasted between 26 and 71 min (mean = 50·44, sd = 12·04) and were audio recorded. The interviews were facilitated by the lead researcher, who utilised a semi-structured interview approach.

### Data collection materials

**Fig. 1:**
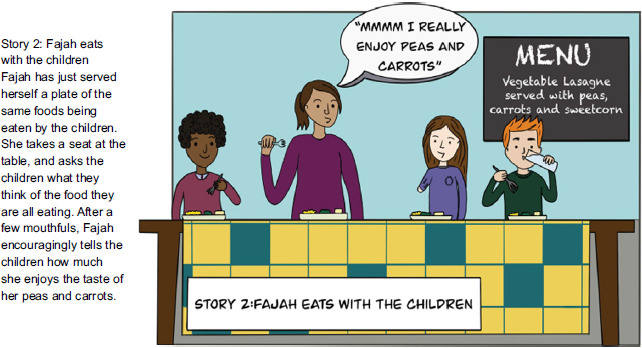
Modelling feeding practice vignette

Four vignettes were designed to present short story scenarios representing each of the four chosen feeding practices: modelling; involvement in food choice; involvement in food preparation and cooking; and involvement in meal planning. Vignettes are used in research as an elicitation tool to stimulate discussion^(35)^ and have been used in other qualitative eating behaviour research^(36)^. Each vignette presented brief written text of a hypothetical story of a feeding practice used by staff with children in a holiday club; these were written by the lead researcher and agreed with the research team. Alongside the written text, pictorial illustrations were presented to enable the participants to visualise the feeding practice scenarios depicted in the stories. The illustrations were designed by LS (see acknowledgements), a researcher known to the research team with skills in designing visual/illustrative content. The vignette content and illustrations were reviewed by CH, CM and EH to enable refinement before use during data collection. Figure 1 provides an example of the text and pictorial content of the modelling feeding practice vignette.

A semi-structured interview schedule (see Table 2) was devised based on relevant academic literature. Interviews began with an icebreaker question (part 1). The researcher then presented and read the first vignette and a subsequent vignette-specific question (part 2a) was asked to ensure the participant had engaged with the vignette and understood what was being depicted. Following this, several further questions were asked (part 2b) specific to the feeding practice depicted. This process was repeated four times until all four vignettes had been discussed, followed by a few broader closing questions (part 3).

**Table 2:** Key interview schedule questions

Part 1: Icebreaker • Please tell me a bit about your role in the holiday club.
Part 2a: Vignette-specific questions • Story 1: Danny gets the kids involved in food preparation and cooking: Why might Danny have invited the children to prepare and cook the food? • Story 2: Fajah eats with the children: Why might Fajah have decided to eat with the children and encouragingly tell them how much she is enjoying her vegetables? • Story 3: The children write the shopping list: Why did Tony and Kesha involve the children in deciding what meals to make and which foods to add to the shopping list? • Story 4- The food buffet is open: Why did Grendon holiday club choose to provide a selection of main meals and salad/vegetables for children to choose from?
Part 2b: Questions presented for each specific vignette discussion • What are your thoughts about this scenario in your holiday club? • Do you have any examples of similar scenarios that you have used in your holiday club? • Could you see yourself and other staff being involved with this type of activity in the holiday club in the future? • What would be the benefits and challenges of using this approach?
Part 3: General and closing questions • Do you have any overall thoughts or comments you would like to share about the different approaches and what or how you might apply these within your holiday club? • Do you have any examples of other ways in which you promote healthy eating in your club? • How could you be better supported to promote healthy eating? • Before we finish, is there anything else you would like to discuss relating to the topics we have been discussing today?

### Data analysis

Interviews were transcribed and analysed using Braun and Clarke’s thematic analysis framework^(37)^. The thematic analysis was conducted using the analysis software NVivo, enabling the data to be organised into generated codes and themes.

A deductive approach was adopted at a higher order data level, using the four dimensions of feasibility previously outlined, to explore the feasibility of staff using the aforementioned feeding practices to promote children’s healthy eating. A bottom-up, inductive approach was also adopted at the lower order data level (themes within the four feasibility dimensions), as there is no feasibility literature available regarding the use of feeding practices in holiday clubs, and therefore the themes generated were data driven. Other feasibility research has adopted this hybrid approach to thematic analysis^(38)^.

The lead researcher (NB) read each transcript to become familiarised with the data. Transcripts were coded, and these codes were organised into an initial thematic framework. Transcripts were further coded and recoded as the coding and thematic framework evolved. An additional researcher (JA) performed coding analysis on 10 % of the transcripts, enabling the lead researcher to compare this coding against their own to assure the lead researcher that they were appropriately interpreting and coding the data. In rare circumstances where there were interpretation differences in the coding, the lead researcher reflected and reconsidered their coding as part of the natural recoding and reflexive process. Additionally, CH, CM and EH reviewed and provided feedback on the thematic framework to improve the data interpretation, structure and robustness.

## Results

### Participant characteristics

The participating club staff (*n* 25) ranged from 23 to 69 years old (mean = 48·08 years, sd = 17·85) and were predominantly female (*n* 18, 72 %) and White British (*n* 23, 92 %). Some staff had a nutrition qualification or training (*n* 8, 32 %), including food hygiene, nutrition for sport/physical activity and nutrition for the catering industry. The number of years working at the club ranged from 1 to 25 years (mean = 4·68 years, sd = 5·40). The roles which staff held were varied, including club leadership (*n* 7, 28 %), project leadership (*n* 4, 16 %), coordinator (*n* 15, 60 %), cooking and catering (*n* 5, 20 %) and coach/youth worker (*n* 2, 8 %). Staff often held dual roles.

### Thematic analysis

**Fig. 2:**
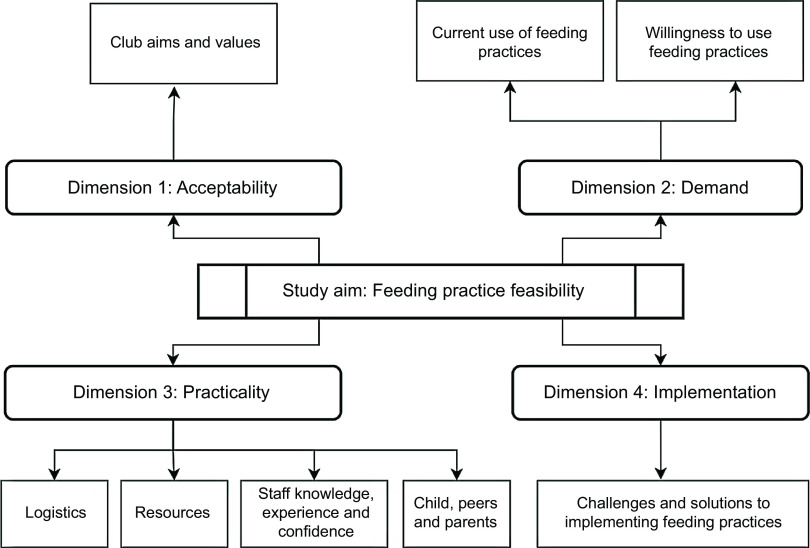
Study themes and feasibility dimensions

Eight themes were identified, which were aligned to the four dimensions of the feasibility framework (acceptability; demand; practicality; implementation)^(31)^. Figure 2 shows the study themes and which feasibility dimension each theme relates to.

#### Dimension 1: Acceptability

Acceptability explores the extent to which a new idea is judged as suitable or attractive^(31)^. One theme relating to the staff acceptability of using the four feeding practices was identified: whether the feeding practices align with the holiday club aims and values.

##### Alignment with club aims and values

Staff reflected on numerous aims and values underpinning the clubs, such as promoting healthy eating, promoting health, well-being and development and focusing on families as well as children. Some staff described how some feeding practices were supportive of achieving their club aims. For example, one member of staff described how involving children in food preparation and cooking would complement their values in encouraging children’s participation in the provision they offer within their club. Similarly, another staff reflected on how involving children in meal planning would support their aim of feeding children because this would *‘make sure there’s something there that everyone likes’* (P6). Another participant described how modelling healthy eating and eating together aligns with their core values of building community and facilitating community eating:

*‘At church, we are part of and we are community and we are one…the church is not dishing out food to you, it is (that) we are genuinely all doing this together.’* (P15)

In other cases, some staff described how some feeding practices may be more challenging to use if they do not complement their core aims of feeding children. For example, for the food preparation and cooking feeding practice:

*‘I had this vision that our club would be very much focused on encouraging the parents and carers to cook with their children (in the club). It became much more of a need to feed the families. So rather than having this club where people came and learned to cook and took their skills home, it very much turned into a meeting the need of people who were struggling to put a healthy meal on the table.’* (P12)

In summary, when feeding practices align with club aims and values, they are more likely to be acceptable to use in the holiday club.

#### Dimension 2: Demand

Demand explores the extent to which a new idea is likely to be used^(31)^. The thematic analysis identified two themes relating to the demand for the four feeding practices: 1. current use of the practice within their club and 2. willingness to use the practice in the future.

##### Current use of the four feeding practices

Many staff who felt they used one or more of the feeding practices discussed various ways each feeding practice was being used. As an example, in the following quote, the staff member provided examples of how they involve children in food preparation and cooking and food choice:

*‘We get them involved with making the lunch. What we would then do with them is put these items on the table in front of the children. So in one of our clubs, we had a buffet, and they could go along and pick anything they wanted. And we used to try and encourage, ‘try a little bit of everything’.* (P21)

The staff member also went on to provide some description of using modelling within their club:

*‘And then, when it was in some schools, it was all on the table and they would help themselves. The staff would have a bit as well. But then we’d ask the children to try it, and help themselves.’* (P21)

In summary, staff reflecting their current use of the feeding practices is an indicator of their ‘demand’ within the holiday clubs.

##### Willingness to use the four feeding practices

Staff reflected on their willingness to use the feeding practices in the future, or use them more proactively if they already use them to some extent. Some staff were already using feeding practices and planned to continue to do so. In other cases, staff were not currently using the feeding practice but were willing to explore using them. For example, for involving children in food choice:

*‘I think it (involving children in choosing their food) would be really worth exploring though, to see, by giving young people autonomy over those choices, over time, I don’t think if you just did it for a week or so, you probably wouldn’t be able to make much change. But if it was something that we could do over at least a 6-month period, so that option was always there every single time, at some point it would probably trigger young people’s autonomous decisions to actually do that.’* (P4)

However, in some cases, staff were not using the feeding practices and explained why they were less willing to use them. For example, for involving children in meal planning:

*‘So my ingredient cost will be 85p for food on the plate. The way we are able to do that is through using surplus food, and with surplus food you just have to use what you’ve got. Financially we just don’t have the luxury of that. We’re always on a shoestring.’* (P17)

In summary, staff’s willingness to use the feeding practices is an indicator of their ‘demand’ for the feeding practices within the holiday clubs.

#### Dimension 3: Practicality

Practicality explores the extent an idea can be carried out with intended participants using existing means, resources and circumstances^(31)^. The thematic analysis identified four themes perceived to be important in determining the practicalities of using the feeding practices, including: logistics; resources; staff knowledge, experience and confidence; children, peers and parents.

Table 3 illustrates the four themes surrounding the factors that staff perceived to be important in determining the practicalities of using the feeding practices, with further explanations provided in the subsequent practicality themes provided.

**Table 3:** Examples of perceived explanatory factors relating to the practicality of using the four feeding practices in holiday clubs

Logistical considerations	Practical to use feeding practices?	
	Yes	No
Factor 1: Logistics		
Amount of work involved	Staff have to do the food shopping anyway (meal planning).	Engaging children in cooking requires a lot of work (food preparation and cooking).
Timings of activities	Staff could involve children in preparing food at the end of the day ready for cooking the following day (food preparation and cooking).	Staff plan the meals during term time ready for the holidays (meal planning).
Frequency of holiday club provision	Staff could get children involved in meal planning one week in readiness for the following week (meal planning).	Club only runs one day per week, making it difficult to involve children in food activities (food preparation and cooking).
Affordability and practicality	Eating with the children is free and easy to implement (modelling).	Staff know what meals are healthy, tasty and within budget, therefore involving children in meal planning is unnecessary (meal planning).
Volume of children attending the club	Staff can break a large group into smaller groups to engage them in cooking activities (food preparation and cooking).	If there is a large number of children to feed, allowing them to make their own choices will take too long to serve the food to children (food choice).
Meal preparation and serving methods	Enabling food choice using a buffet serving style will be easy to implement (food choice).	Food menus are fixed and organised by the school meal service (meal planning).
Health, safety and dietary considerations	Sensible to provide a range of choices to cater for different dietary needs (food choice).	Too many health and safety protocols to consider when involving children in cooking (food preparation and cooking).
Factor 2: Resources		
Finances	Staff have the financial resources to underpin the feeding practice implementation (no specific feeding practice).	Staff have difficulty accessing funding as the grant process is challenging (no specific feeding practice).
Time	Lots of choice is unfeasible, but a couple of options are possible (food choice).	Cooking takes much longer when involving children (food preparation and cooking).
Staffing and volunteering	There are sufficient numbers of staff to sit and eat with the children (modelling).	Club has inconsistent staffing, making it difficult to implement feeding practices consistently (no specific feeding practice).
Facilities	Staff have the equipment needed to involve children in cooking activities (food preparation and cooking).	Difficult to access kitchen facilities as club is run in a shared community hall (food preparation and cooking).
Partnership working	Food partners receptive to providing fresh vegetables for children to prepare (food preparation and cooking).	Staff do not know what surplus food they will receive, and it often has a short shelf-life, making it difficult to involve children in meal planning (meal planning).
Factor 3: Staff knowledge, experience and confidence		
Knowledge, experience and confidence	Staff have the relevant skills in working with children to be effective role models (modelling).	Staff do not feel confident involving children in cooking activities (food preparation and cooking).
Importance of training	Staff are provided training in food hygiene, safeguarding and first aid to enable them to handle food and involve children in cooking (food preparation and cooking).	A lack of training may result in role modelling negative eating behaviour (modelling).
Factor 4: Children, peers and parents		
Age of the children	Modelling healthy eating may be more successful with younger children (modelling).	Meal planning and shopping are difficult with younger children (meal planning).
Children’s preferences and responses to food and food activities	Many children enjoy being involved in cooking (food preparation and cooking).	If children are able to make choices about food, they will choose unhealthy options (meal planning and food choice).
Peer influence	Peers are effective role models as they build a rapport with children that adults cannot (modelling).	If one child refuses a food or activity, other children may do the same (no specific feeding practice).
Parent influence	Parental modelling of healthy eating often encourages their child to do the same (modelling).	Parents may pressure their child to eat or avoid eating certain foods (food choice).

##### Logistics

Staff reflected various logistical factors influencing the perceived practicality of using the feeding practices. These are listed in Table 3, which provides examples of where logistical factors can make using the feeding practices practical or impractical. Some staff felt these logistical factors could be overcome, and therefore it would be practical to use the feeding practices:

*‘The only challenges that I can see would be health and safety challenges, which I think are quite easily avoided.’* (P2, food preparation and cooking)

Other staff felt that these factors would make the feeding practices impractical to use:

*‘We have only about half an hour before they eat. If we were leaving veg prep until the last minute, I think our kitchen team would have a heart attack thinking about whether they could do it (involve children in food preparation and cooking) or not.’* (P9, food preparation and cooking)

##### Resources

Staff identified resources needed to make it practical for them to use the feeding practices, including finances, time, facilities and equipment, partnership working and staffing (Table 3). Some staff reflected on how a lack of resources would challenge the practicality of using the feeding practices, while other staff had sufficient resources to support using the feeding practices. For example, when involving children in food preparation and cooking activities:

*‘We would have a problem involving the children in the kitchen, because it’s not our space, because it’s a small space…With the restrictive space and with the equipment that doesn’t belong to us, with a lot of hot equipment, I think the scenario depicted here wouldn’t work.’* (P19)

##### Staff knowledge, experience and confidence

Staff reflected how the knowledge, experience and confidence of staff influenced the practicality of using the feeding practices (Table 3). Most staff demonstrated a good knowledge and understanding of the importance of using the feeding practices to support positive outcomes for children. For example, the following member of staff reflected on various positive outcomes from involving children in food preparation and cooking activities, including providing new experiences for children and the short- and long-term implications of providing educational opportunities about food:

*‘I would think that’s about education, isn’t it? It’s that thing of, if you give a man a fish, he could feed himself that day, but if you give him the skills then you set them up for life. And actually it’s about, it can be about changing behaviours and about bringing about enjoyment of preparing meals so that they then take a healthy interest into their adult life. So it’s about developing healthy habits in a fun way. And it may be an experience that they are not getting at home with parents, because parents aren’t always very keen to have their children in the kitchen, and actually a lot of parents haven’t got the skills themselves. So it’s about breaking those cycles and the behaviours.’* (P18)

In addition to knowledge about the feeding practices, staff come from various backgrounds such as youth work, education, food and nutrition, which influenced staff experience and their perceived capability to use the feeding practices. For example, for modelling, some staff felt they had the relevant previous experience to be positive role models of food to children:

*‘We are quite lucky, in previous years we’ve had lots of really good people…retired ex-professionals and things like that, that are used to working with families and children. So I think that it would go down well actually.’* (P6)

Some staff indicated lack of experience as a barrier to role model eating behaviours to children:

*‘Some volunteers are more confident than others about sitting with families and just chatting with them…if it’s your first-time volunteering, and you don’t perhaps know people at all, not all our volunteers are as confident as the rest of them.’* (P22)

Many staff emphasised the importance of providing training opportunities for staff such as food hygiene, safeguarding, play training and healthy eating, to ensure staff have the skills and confidence to use the feeding practices with children:

*‘Training staff up, making sure they’ve got all the qualifications, or recruiting new parents and volunteers. It’s all obviously a major factor into how the sessions are delivered, because it can’t always be the same staff that are at each of the sessions.’* (P7)

##### Children, peers and parents

Staff reflected on the impact of child factors such as age, children’s food preferences and responses to food and food activities on the practicality of using the feeding practices (Table 3). For example, some staff felt that involving children in food preparation and cooking was something that *‘would work with the older children’*, while other staff felt that ‘*cooking is something that crosses all age groups, and even the youngest, toddlers, loved getting involved in the kitchen and making a bit of a mess*.’ (P10)

Staff reported that food preferences and responses to food among children, from enjoyment and curiosity among some children, and fussiness and food refusal among other children influenced staff perceptions of the practicality of using the feeding practices. For example, when involving children in making food choices, staff often felt that this provided opportunities for children to select foods they will enjoy while also exposing a risk of foods being refused and therefore wasted:

*‘It’s difficult, you don’t want to encourage fussy eating, do you? That’s difficult because you really want to encourage healthy eating, and if you can broaden their menu then that’s what you really want. But equally, you don’t want to put something in front of them then them say ‘well I’m not eating that’.’* (P20)

Staff also reflected on how peers and parents can have an influential role in how practical it is to use the feeding practices. For example, when offering food choice:

*‘Sometimes some of the younger children will sort of shy away a bit and look to mum, and that can go either of two ways. Mum can either go ‘oh yes just try a little bit’, or “no he won’t eat that”.’* (P22)

#### Dimension 4: Implementation

Implementation explores whether a new idea can be successfully delivered to intended participants in some defined, but not fully controlled, context^(31)^. As described in the practicality dimension, staff identified various logistical and resource challenges to implementing the feeding practices, although some staff believed these challenges could be overcome. Table 4 provides examples of the challenges along with examples of adaptations and solutions that staff identified to implement the feeding practices.

**Table 4:** Challenges and solutions to implementing feeding practices in holiday clubs

Challenge type	Challenges	Solutions
Modelling		
Logistics Resources	Staff have multiple responsibilities including cooking and serving food and keeping children seated which makes it difficult to sit and eat with the children. Staff do not know how many children will arrive at the club, and it can be more difficult for staff to sit with the children during mealtimes when the club is busy.	• Recruit more staff or volunteers where possible. • Encourage older children and parents to act as models of healthy eating.
Offering choice		
Logistics	It is difficult to involve children in choosing their own foods using a buffet-style serving methods when there is a large number of children as it will take too long to serve the food.	• Buffet style serving method could be replaced with family style dining, where food options are placed on the table for children to choose from.
Resources	Staff do not have the kitchen facilities and staffing to enable them to provide a range of food options for children, but can offer accompaniments at the table for children to choose from.	• Main dish could be served to children but accompaniments could be provided at the table for children to choose from.
Resources	Staff lack the time and resources to provide a range of different food options for children to choose from.	• Two food options could be provided to offer a level of choice for children.
Logistics	As food is batch cooked and frozen in preparation for the club provision, staff already know what they are planning to provide children at each mealtime.	• Staff could ask the children if they would like to request a meal to be served from the list of meals that have been batch cooked and frozen.
Food preparation and cooking		
Resources	Club food provision is reliant on food surplus donations, which are usually preprepared food items and therefore do not facilitate involving children in food preparation and cooking activities.	• Explore additional local food partnerships who could donate fresh food items to involve children in basic food activities.
Resources	Clubs lacks the necessary cooking facilities to involve children in meal preparation and cooking activities.	• Involve children in basic food activities such as chopping and building (e.g. making desserts, building fruit skewers) rather than full cooking activities.
Logistics	There is a large amount of work involved and health and safety considerations in engaging children in food preparation and activities (e.g. creates a lot of washing up).	• Staff prepare the foods and the children then build their own foods (e.g. tortilla wraps). • Provide safe knives to enable children to prepare foods safely.
Logistics	Club only runs one day per week of each holiday, making it difficult to frequently involve regular children in food preparation and cooking. Food is batch cooked and frozen during school term time in readiness for the holiday club as this is an affordable and practical model of working, making it difficult to involve children in the cooking of the food.	• Involve children in food preparation and cooking as additional food activities (e.g. cooking workshops to prepare food to take home at the end of the day) during the club day, rather than preparing for the meal served during club mealtimes. • Provide an activity table where children can informally chop and prepare foods as part of the broader club activity.
Logistics	It is difficult to involve children in food preparation and cooking activities when there are a large volume of children attending the club.	• Involve only a small number of children at a time in food preparation activities or split larger groups up into smaller groups (ensures children get the support they need and do not struggle with the activities).
Meal planning and writing shopping lists		
Logistics	• Meals are planned during the school term in readiness for the holiday period, making it difficult to involve children in meal planning activities. • Food is batch cooked and frozen in advance of providing to holiday clubs, making it difficult to involve children in meal planning and writing shopping lists.	• Children could be involved in meal planning at the end of the club period in readiness for future club provision.
Logistics	• Being able to involve children in meal planning depends on how frequently the holiday club runs. For example, some holiday clubs only run for 1·5–2 h per week of each holiday period, and some only run one day per week of each holiday periods, making it difficult to frequently involve children in meal planning and writing shopping lists.	• Staff could involve children in meal planning and writing shopping lists at the end of the holiday club session and plan their meals for the following session.
Logistics Resources	• Food menus are organised by the school meal service/public health teams, making it difficult to involve children in meal planning activities. • Staff do not always know what food surplus donations they are going to receive from their food partners, which sometimes makes their meal planning unpredictable and last minute and therefore difficult to involve children in meal planning.	• Staff could involve children in meal planning and writing shopping lists as a general activity of its own rather than planning specifically for the meals to be provided within the club.
Logistics	• Children have different ideas, preferences and dietary needs, and therefore will likely come up with a range of different meal options, making it difficult to involve children in meal planning activities. This is particularly challenging when working with a large volume of children. • Children will likely request unhealthy food options if they are given the opportunity to meal plan.	• Staff can provide a couple or a small list of (healthy) meal options and enable children to choose the meals from the list and/or ask the children what ingredients would be needed to make the meals. • Staff can split groups up into smaller groups in order to make meal planning activities more manageable.

## Discussion

This study explored staff perspectives of the feasibility of using four evidence-based feeding practices (modelling; involvement in food choice; involvement in food preparation and cooking; involvement in meal planning) to promote healthy eating in holiday clubs among children from food insecure backgrounds. The findings revealed that the feasibility of using the feeding practices varied between staff and between feeding practices. This is unsurprising given that holiday clubs vary in how they operate, who they target and what provision they offer^(39)^. Using four dimensions of a feasibility framework^(31)^, the thematic analysis revealed that the feeding practices demonstrated good acceptability (feasibility dimension 1), reinforced through one theme: the feeding practices alignment with club aims and values. Staff also reported good demand (feasibility dimension 2) for the feeding practices, reinforced through two themes: current use and willingness to use the feeding practices in the future. Staff perceived that the practicality (feasibility dimension 3) of the feeding practices was dependent on numerous factors (logistics, resources, staff readiness, children, peers and parents). However, in the fourth feasibility dimension (implementation), staff provided various practical solutions to overcome perceived and actual barriers identified under ‘practicality’. These findings offer novel insights into the application of evidence-based feeding practices in holiday club settings to promote children’s healthy eating.

Staff acceptability of using the four feeding practices was demonstrated through their perceptions related to how the feeding practices align with the club aims and values. Previous research suggests that organisational aims, values and culture are important factors influencing the success of health promotion and nutrition interventions in childcare settings^(40)^. The links that staff in this current study made between the feeding practices and their core organisational aims are therefore promising findings in this study. However, it is important to note that a core priority for the staff was ensuring children are fed within the club; in circumstances where other factors such as children’s fussy eating, children’s food preferences and resource constraints are experienced, this could require clubs to prioritise the provision of food ahead of their healthy eating agendas. While this is logical and necessary from a human need perspective, children from already disadvantaged backgrounds may miss opportunities to be exposed to a more varied repertoire of foods and food experiences that promote nutritional, social and educational experiences for children. From a practical perspective, families experiencing food insecurity may not have sufficient resources to buy and offer new foods which children might refuse^(6)^, and holiday clubs are only able to offer foods that they have/received, both of which can limit children’s exposure to a variety of food types. Additionally, from a nutritional and educational perspective, this highlights the importance of also drawing on a range of other feeding practices beyond the four explored in this study; for example, holiday clubs could increase children’s nutrition through concealing healthy food within liked/familiar foods (e.g. inside sauces), provided children are also exposed to healthy foods in identifiable forms as this is essential for promoting children’s educational awareness, liking and familiarity of these foods^(41)^.

Staff demand for the four feeding practices was demonstrated through their current use of and willingness to use the feeding practices in the future. The staff generally showed a high level of receptivity to using the feeding practices in the future, or using them more proactively if they already used these to some degree. These findings are highly promising as they demonstrate a demand to use the feeding practices; ‘demand’ is an important area of focus in feasibility studies, the actual use or expressed interest or intention to use an intervention (in this case, the use of the four feeding practices) increases the likelihood of the intervention being used in the future^(31)^. There were examples, however, where staff were less willing to explore the feeding practices in the future; this was typically interlinked with staff perceptions of the practicality to use the feeding practices with children. Existing research related to promoting children’s healthy eating reports numerous barriers to implementing feeding practices. For example, barriers to parents reoffering vegetables to their children include concerns around time, food waste, children’s mood and having tantrums^(42)^. In this current study, the reluctance to implement the feeding practices emphasises the importance of offering staff information and training resources to highlight various ways the feeding practices can be meaningfully implemented. However, the findings are overall promising as they mostly reflect a general demand for feeding practices, where staff demonstrated examples of good practice already being implemented in holiday clubs to promote healthy eating, and many expressed willingness to explore the use of the feeding practices in the future.

Although staff generally demonstrated good acceptability and demand for the four feeding practices, there were various logistical, resource, staff, child and parent factors influencing staff perceptions of the practicality of using the feeding practices in their holiday clubs. These factors were described as opportunities to use the feeding practices in some clubs and barriers in others. Previous research suggests that providing food, using feeding practices and promoting healthy eating require the procurement of sufficient resources such as staffing, funding, facilities and partnership working^(27,40)^. The results of this current study illustrate that while holiday clubs face a diverse range of challenges, there are often workable solutions or adaptations that enable staff to implement the feeding practices. These findings echo those from research outside of holiday clubs related to promoting children’s healthy eating. For example, a qualitative study exploring parent barriers to promoting children’s healthy eating identified that while parents face numerous challenges (lack of time and resources, and children’s food preferences/picking eating), there may also be strategies available to overcome these challenges (e.g. selecting healthy meals that are quick to prepare, buying healthy foods that are on sale)^(43)^. It should be noted that their study was conducted with families from a middle-class background, and therefore results may vary among families from disadvantaged communities as the barriers for these parents to promote children’s healthy eating are often magnified. For example, families experiencing food insecurity may have to purchase cheaper foods, which are commonly less nutritious^(6)^, and they may be more likely to use more controlling feeding practices, like pressuring children to eat, as a way to ensure that children are fed and to avoid food waste, given the limited resources and sources of food available^(7)^.

The findings of this current study reinforce that, to enable staff to identify simple and flexible ways to implement feeding practices, information and training resources should be provided which highlight different ways that evidence-based feeding practices can be achievably implemented within and around numerous logistical and resource constraints faced in holiday clubs. Best practice examples could usefully be shared across holiday club networks to achieve buy-in at the programme as well as individual level. These information, training and sharing resources also have policy applications, as they align with the healthy eating public health priority of the UK’s current 2020–2025 Public Health Strategy^(44)^.

The HAF programme is a national UK government funded programme aiming to provide food (to promote healthy eating), activity (to promote exercise), and enrichment (to promote socialising, expression and creativity) opportunities through new and existing UK holiday clubs to children who are FSM eligible^(22)^. The HAF programme has recently embedded into its programme standards a mandatory requirement for holiday clubs to work towards providing enrichment opportunities to children, including feeding practices such as nutrition education and involvement in food-related activities^(22,28)^. The HAF programme would therefore provide a useful platform to share resources and best practice. The UK government has increased the HAF programme considerably, from £9million in 2020 across ten UK geographic areas and 50 000 FSM eligible children^(45)^, to £220million in 2021 to holiday clubs across the country^(46)^. While this funding is considerable, this current study highlights that many holiday clubs are still operating with minimal, time-limited resources. As of October 2021, the government pledged to invest over £200million per year for three years. For the HAF programme^(22)^; while this is promising, longer-term funding plans would enable holiday clubs to more effectively plan their long-term delivery strategy.

The study findings should be considered alongside some methodological strengths and challenges. Feasibility studies aim to understand how to implement a programme within the constraints faced within the population^(31)^. This aim was achieved in this feasibility study, because although staff highlighted numerous perceived barriers to using evidence-based feeding practices to promote children’s healthy eating behaviours, the findings showed many of the barriers were surmountable. A further strength is that qualitative methods are recommended to assess feasibility of behavioural and social interventions, as they generate understanding of how the target population will receive the intervention and the factors facilitating or undermining its delivery^(30)^. This study was of particular value because it provided a platform for staff to identify real-world examples of how to feasibly implement feeding practices around the facilitators and barriers that exist in holiday club settings. Such stakeholder cocreation is a key facet of intervention development. Moreover, using the vignettes alongside interviews allows exploration of complex health issues such as promoting healthy eating, as they provide structure to interviews while enabling participants to respond in greater depth^(36)^.

An inherent challenge with qualitative research is low sample sizes, which may limit the generalisability of the study findings to the wider population^(47)^. However, large sample sizes and generalisability are not the goal of qualitative research. Rather, qualitative research seeks to develop rich and detailed understanding of peoples thoughts, insights and experiences related to the research topic of interest^(48)^. Indeed, this study engaged 25 staff from 24 holiday clubs implementing various local and national holiday club programmes and gained rich insights about their perceptions of the feasibility to use evidence-based feeding practices to promote children’s healthy eating in holiday clubs. Moreover, the twenty-five staff involved from the twenty-four holiday clubs were located in nine UK regions, and therefore the results reflect the perceptions of staff from a range of holiday clubs across the UK. However, the study did not manage to recruit club staff from the North of England, and it is possible that staff experiences may vary in this region. It is particularly important to note this limitation given that the North East is among the most deprived regions in England^(49)^.

Social desirability is also a consideration relevant to this study. Social desirability bias may occur when using qualitative methods for feasibility research^(38)^, and indeed when using vignettes, where participants may over-report positive perceptions and under-report negative perceptions about the study topics being explored^(38)^. However, the study data found a range of different perceptions and experiences from the participants, and participants appeared reflective and engaged throughout the interview process. This provides confidence that participants were able to share their perceptions honestly, limiting social desirability bias.

The interviews conducted for this study were video-based, as they were completed during the COVID-19 pandemic where social distancing measures were required. The advantages and disadvantages of conducting interviews using video platforms are well reported elsewhere^(50)^. A benefit of using a video-based platform was in its screen-sharing functionality, which enabled the researcher and the participant to view and reflect the vignettes in real-time during the interview, rather than viewing them independently which would be more detached and impersonal during the interview process.

In summary, this study revealed that the feasibility of using four evidence-based feeding practices (modelling; involvement in food choice; involvement in food preparation and cooking; involvement in meal planning) to promote children’s healthy eating in UK holiday clubs varied between staff and between feeding practices. Nevertheless, staff generally demonstrated acceptability and demand for evidence-based feeding practices, but there were concerns expressed the around practicality of implementation. However, across the study participants, staff provided examples of simple solutions or adaptations that could be made to enable staff to implement the feeding practices. These findings highlight the importance of training and sharing resources and best practice to support staff to achievably implement evidence-based feeding practices. Future research is needed to codesign resources with holiday club staff to ensure that they are maximally beneficial and to explore the impact of feeding practice interventions on staff knowledge, experience, confidence and use of such practices to promote children’s healthy eating behaviours. Future research would also be meaningful with broader stakeholders of holiday clubs, including the programme managers, parents and children attending the holiday clubs, to explore their perspectives of the impact of positive feeding practices on children’s eating behaviour outcomes such as their consumption of and their willingness to try novel and healthy foods. As the first known study to explore the feasibility of implementing feeding practices into holiday club feeding routines, the study findings offer meaningful insights into the potential for holiday clubs to utilise evidence-based feeding practices to support and empower clubs to maximise their opportunities to promote healthy eating among children at risk of food insecurity.
